# Bio-Functional Natural Products in Edible Resources for Human Health and Beauty

**DOI:** 10.3390/molecules27165060

**Published:** 2022-08-10

**Authors:** Toshio Morikawa

**Affiliations:** Pharmaceutical Research and Technology Institute, Kindai University, 3-4-1 Kowakae, Higashiosaka 577-8502, Osaka, Japan; morikawa@kindai.ac.jp

Natural products remain important repositories of promising therapeutic candidates due to their rich chemical and biological diversity. In particular, the development and application of bio-functional natural products from edible resources for the prevention of human diseases, health maintenance, and beauty are attractive for practical research.

The Special Issue “Bio-functional Natural Products in Edible Resources for Human Health and Beauty”, published in the journal *Molecules*, includes twelve articles, including ten original and two review articles.

The original papers include ‘Anthocyanin accumulation in the leaves of the purple sweet potato (*Ipomoea batatas* L.) cultivars’ by Guo Liang Li et al. [1]; ‘Composition of sugars in wild and cultivated lingonberries (*Vaccinium vitis-ideaea* L.)’ by Gabriele Vilkickyte et al. [2]; ‘NO_x_-, IL-1β-, TNF-α-, and IL-6-inhibiting effects and trypanocidal activity of banana (*Musa acuminata*) bracts and flowers: UPLC-HRESI-MS detection of phenylpropanoid sucrose esters’ by Louis P. Sandjo et al. [3]; A new eucalyptol-rich lavender (*Lavandula stoechas* L.) essential oil: emerging potential for therapy against inflammation and cancer’ by Mohamed Nadjib Boukhatem et al. [4]; ‘Natural herbal estrogen-mimetics (phytoestrogens) promote the differentiation of fallopian tube epithelium into multi-ciliated cells via estrogen receptor beta’ by Maobi Zhu et al. [5]; ‘Sea buckthorn leaf powders: the impact of cultivar and drying mode on antioxidant, phytochemical, and chromatic profile of valuable resource’ by Lina Raudone et al. [6]; ‘Co-treatments of edible curcumin from turmeric rhizomes and chemotherapeutic drugs on cytotoxicity and FLT3 protein expression in leukemic stem cells’ by Fah Chueahongthong et al. [7]; ‘Lycoperoside H, a tomato seed saponin, improves epidermal dehydration by increasing ceramide in the stratum corneum and steroidal anti-inflammatory effects’ by Shogo Takeda et al. [8]; ‘Consumption of sinlek rice drink improved red cells indices in anemic elderly subjects’ by Peerasak Lerttrakarnnon et al. [9]; and ‘Stress-relieving effects of sesame oil aroma and identification of the active components’ by Hiroaki Takemoto et al. [10].

In addition, two review papers are included, namely ‘Natural ingredients from medicine food homology as chemopreventive reagents against type 2 diabetes mellitus by modulating gut microbiota homoeostasis’ by Xiaoyan Xia and Jiao Xiao [11] and ‘Phytotherapeutic approaches to the prevention of age-related changes and the extension of active longevity’, by Olga Babich et al. [12].

As the guest editor of this Special Issue, I hope “Bio-functional Natural Products in Edible Plant for Human Health and Beauty”, appearing in the Natural Products Chemistry section of *Molecules*, will be of use to many researchers. I would like to acknowledge all the authors for their valuable contributions and the reviewers for their constructive remarks. Special thanks to the publishing staff of *Molecules* at MDPI for their professional support in all aspects of this Special Issue.
